# Malnutrition, anemia, micronutrient deficiency and parasitic infections among schoolchildren in rural Tanzania

**DOI:** 10.1371/journal.pntd.0010261

**Published:** 2022-03-04

**Authors:** Emmanuel C. Mrimi, Marta S. Palmeirim, Elihaika G. Minja, Kurt Z. Long, Jennifer Keiser

**Affiliations:** 1 Swiss Tropical and Public Health Institute, Allschwil, Switzerland; 2 University of Basel, Basel, Switzerland; 3 Ifakara Health Institute, Ifakara, Tanzania; University of Texas Medical Branch, UNITED STATES

## Abstract

**Background:**

Malnutrition, anemia, micronutrient deficiency and parasitic infections continue to impact the nutritional status and health of children in lower-income countries. However, not enough data concerning this issue is available. The aim of this study was to assess the distribution of nutritional indicators, anemia and micronutrient deficiency and their underlying risk factors among schoolchildren in south-eastern Tanzania.

**Methodology/Principal findings:**

This cross-sectional study enrolled primary schoolchildren aged 6–12 years from Kikwawila and Kiberege wards, Tanzania. In total, 471 schoolchildren underwent a physical examination and provided blood, stool and urine samples for an assessment of the levels of different micronutrients, nutritional and anemia status, and parasitic infection status. We employed bivariate and multivariate logistic regression to determine the association between nutritional statuses, anemia, micronutrient deficiency and parasitic infections. We found that 23.90%, 12.60% and 16.20% of schoolchildren were stunted, underweight and wasted, respectively. About 14.0% of schoolchildren were found to be anemic. Children diagnosed with *Plasmodium falciparum* infection were more likely to have low levels of ferritin (aOR: 10.40, 95% CI: 2.88-40.53) and elevated levels of serum soluble transferrin receptor (aOR: 3.59, 95% CI: 1.27-11.23), respectively. Vitamin A (34.71%) and vitamin B12 (8.79%) were the most prevalent micronutrients found to be deficient in diagnosed children. Finally, we found that schoolchildren attending the most rural schools were five times more likely to be diagnosed with at least one micronutrient deficiency (aOR: 5.04, 95% CI: 2.38–11.44).

**Conclusions/Significance:**

Malnutrition, anemia and micronutrient deficiency still pose a significant health burden among schoolchildren living in rural Tanzania. To effectively tackle this burden, health interventions such as deworming, micronutrient supplementation, vector control, health education and access to clean water and improved sanitation should be strengthened and made sustainable.

## Author summary

In this study, we assessed the distribution and risk factors of nutritional indicators, anemia and micronutrient deficiency among schoolchildren of the Kikwawila and Kiberege wards, Tanzania. Our analysis revealed that malnutrition in form of stunting and wasting is still very high among schoolchildren in this region. About 14.0% of children were found to be anemic. Micronutrient deficiency was highly variable ranging from 0.50% (Folate) to 35.0% (Vitamin A). Schoolchildren from most rural schools were more likely to be found stunted and deficient with at least one type of micronutrient. Efforts to control and eliminate malnutrition, anemia, micronutrient deficiency and parasitic infections would benefit from sustainable and integrated approaches such as deworming, micronutrient supplementation, health education, vector control and an improvement in access to clean water and improved sanitation.

## Introduction

Malnutrition is a significant global public health problem that encompasses undernutrition, overweight, obesity and diet-related non-communicable diseases [1]. In Sub-Saharan Africa, children and women of reproductive age bear the highest burden of malnutrition, parasitic infections and micronutrient deficiency. The high prevalence of stunting (50%) and underweight (25%) in children under five years of age in this region is staggering [2,3]. Parasitic infections play a crucial role in increasing malnutrition by compromising the immune system and altering the macro- and/or micronutrient balance of the body [4]. Additionally, infections with *Giardia lamblia*, coccidia, *Plasmodium* spp., *Schistosoma* spp. and soil-transmitted helminths have been shown to alter the nutrient pools by affecting food intake, metabolism and the micronutrient uptake in the gut [5]. Poor uptake of nutrients can lead to a micronutrient deficiency, which is another form of undernutrition caused by lack of essential vitamins and minerals in the body [1]. During childhood, lack of these essentials vitamins and minerals can directly affect growth and development, morbidity and the chances of survival of a child [6].

Micronutrient deficiencies include lack of iron, vitamin A and iodine, which put more than two billion people at risk worldwide by causing anemia, night blindness and goitre diseases, respectively [6]. Iron deficiency is the most common micronutrient deficiency and cause of anemia worldwide [7]. Among children younger than five years of age, approximately 42% of anemia is attributable to iron deficiency [8]. Vitamin A deficiency has also been shown to cause anemia, although its effect is more likely to cause night blindness [9,10]. A higher dosage of oral-vitamin A supplementation (≥ 30 mg) to infants in areas with a high prevalence of deficiency, as recommended by the World Health Organization (WHO), has resulted in a 10% reduction in prevalence of vitamin A deficiency [11,12]. Despite these efforts, vitamin A deficiency still disproportionately affects poor societies from lower-income countries, where more than a quarter of the malnourished population is considered vitamin A deficient [13,14].

Data pertaining to the prevalence of parasitic infections and its effect on nutritional status, anemia and micronutrient levels on schoolchildren are scarce in resource-limited settings, including Tanzania. A longitudinal study undertaken by Tanner and colleagues from 1982 to 1984 [15], was the last survey that attempted to determine the relationship between parasitic infections and nutritional variables in south-eastern Tanzania. The aim of the present study was to investigate the macro-nutritional and anemia status of schoolchildren from the Kikwawila and Kiberege wards in south-eastern Tanzania, as well as these children’s micronutrient concentrations, which has long been overdue. Secondly, we investigated the associations of underlying risk factors with these outcomes. Finally, we explored potential associations of nutritional status, anemia and micronutrient deficiency with parasitic infections, which were previously reported elsewhere [16].

## Methods

### Ethics statement

Ethical clearance for this cross-sectional study was provided by the National Institute of Medical Research (NIMR; reference no. NIMR/HQ/R.8a/Vol. IX/3030), by the Institutional Review Board of the Ifakara Health Institute (IRB-IHI; reference no. IHI/IRB/No: 017–2018) in the United Republic of Tanzania, and by the Ethikkommission Nordwest- und Zentralschweiz (EKNZ; reference no. 2018–00823) in Switzerland. Caregivers of selected schoolchildren were invited for an information session that took place in the nearest participating school. Investigators informed caregivers about the study objectives, procedures, benefits, potential risks and encouraged them to clarify any questions they may have. All caregivers where invited to provide their consent for the enrolment of their child in the study by signing an informed consent form. Caregivers who could not read provided a thumbprint, while an impartial witness who could read and write signed the consent form, confirming that all the relevant information was adequately conveyed to the caregiver. On the following day, all schoolchildren whose parents had given a written consent were summoned and provided with an explanation about the study objectives, procedures, benefits, potential risks and provided with clarification to all of their questions. Each child was then invited to provide an oral assent.

### Study design and setting

This analysis of malnutrition, anemia and micronutrients was part of a cross-sectional study conducted in schoolchildren attending the Lungongole, Katrin, Kapolo, Kibaoni, Kilama A, Kilama B, Kikwawila, Milola and Site primary schools in the Kibaoni and Kiberege wards in south-eastern Tanzania. This cross-sectional study was designed to estimate the parasitic prevalence among 550 randomly selected primary schoolchildren from Kiberege and Kibaoni wards, with a precision limit of 4.00% which is defined as one-half length of the 95.00% confidence interval (CI). Each school was associated with one village. We categorized schools into two groups: most rural and least rural; the most rural schools were located more than 10 km radius away from Ifakara town (the largest town near our study site) and the least rural within less than 10 km radius.

Kibaoni and Kiberege wards are located in the Kilombero valley at an altitude ranging from 270 to 1,000 m above sea level. Temperature and rainfall are relatively high in this region (1,200–1,800 mm and 25°C-32°C, respectively). This study was conducted between April and December 2019, overlapping both dry and rainy seasons. During the rainy season, from November to May, many households in Kikwawila, Lungongole, Kilama A, Kilama B and Katrin villages are not accessible by motor vehicle. The main economic activity in this region is subsistence farming, especially rice, maize, cassava and banana [17]. Farmers also engage in small-scale fishing, hunting and pastoral livestock keeping [17].

### Data and sample collection

Details on the procedures of the entire study, including the physical and clinical examination of participants, collection and analysis of the stool and urine samples, and collection of all demographic, socio-economic status, household characteristics and animal possession data were presented in our previous publication [16]. In brief, enrolled schoolchildren provided at least one stool sample, one urine sample and one venous blood sample. Stool samples were collected in the morning after handing empty containers the previous day. Each of the laboratory techniques used to analyse these samples is described in detail elsewhere [16]. Stool samples were meant to be used to detect infections with intestinal protozoa (via formal-ether concentration technique), *Strongyloides stercoralis* (via Baermann technique) and soil-transmitted helminths (via Kato-Katz technique). However, it was only possible to analyse stool samples for intestinal protozoa since the local health authorities unexpectedly treated all school-aged children in the region with albendazole, an anthelminthic drug, three weeks prior to the start of our sample collection. Urine samples were used to detect infections with *Schistosoma mansoni* (via POC-CCA) and *Schistosoma haematobium* (via urine filtration). The venous blood sample was used to detect infections with *Plasmodium* spp. (via a rapid diagnostic test, and a thick and thin Giemsa smears when a positive results was found with the rapid diagnostic test) and lymphatic filiriasis (via a rapid diagnostic test). Additionally, blood samples were used to conduct a full blood picture and to measure levels of different micronutrients, as described in detail below.

The clinical examination included palpation for liver and spleen enlargement, physical examination of the abdomen, skin, lymph nodes, and pulse and heart rates assessments. After the clinical examination, each student was asked to provide a urine sample in a urine container on the spot and a venous blood sample was taken using intravenous catheter attached to vacutainer tubes (BD Vacutainer). Socio-economic status was assessed using a structured questionnaire of specific household assets (soap, radio, television, cell phone, refrigerator, fan, bicycle, car, tractor and electricity). This questionnaire also collected data on participants’ animal keeping (chicken, pig, cattle, dog and cat), household conditions (type of roof, wall and floor), source of drinking water and presence and location of latrines was recorded.

For anthropometric measurements, a mechanical weight scale (Seca, Switzerland) was used to measure children’s weight (to the nearest 0.1 kg) (S1 Text). A wall mounted roll-ruler stadiometer (Axiom, Germany) was used for height measurement (to the nearest 0.1 cm). The same weight and height scales were used to determine children anthropometry in all primary schools. The body mass index (BMI) was calculated as the ratio of the weight (kg) to the squared height (cm). Weight, height and BMI values were related to age and compared with the WHO child growth standards for weight-for-age (underweight; only for children <10 years), height-for-age (stunting) and BMI-for-age z-scores (wasting).

For the micronutrients analysis, each participant provided a venous blood sample from the antecubital arm vein through an intravenous catheter into two separate tubes: a serum vacutainer tubes (about 7 ml; BD Vacutainer) and a tube containing Na2-EDTA (about 1 ml; Sigma, St. Louis, MO). All samples were cold-chain transported to the Ifakara Health Institute laboratory. Within six hours of collection, blood samples from Na2-EDTA tubes underwent a complete blood count analysis to determine the red blood cell indices, such as mean corpuscular hemoglobin (MCH), mean corpuscular hemoglobin concentration (MCHC) and mean corpuscular volume (MCV). Blood samples from the serum vacutainer tubes were centrifuged to obtain serum and stored at -20°C until they were shipped to Basel, Switzerland. Schoolchildren were not asked to fast or eat before blood sample collection.

### Determination of concentrations of micronutrients

The following micronutrients and their markers were analysed: ferritin, folate, retinol-binding protein (RBP), serum soluble transferrin receptors (sTfR), transferrin, vitamin A, vitamin B12 and zinc. In addition, two inflammatory markers were studied: α-glycoprotein (AGP) and C-reactive protein (CRP). Measurements of micronutrient concentrations were carried out in two laboratories. In the Rothen laboratory (Basel, Switzerland) folate, vitamin A, vitamin B12 and zinc serum levels were determined by chemiluminescence immunoassay (UniCel DxI 800), and transferrin with the turbidimetric procedure (Beckman Coulter AU 680 analyzer). In the VitA-Iron Tech laboratories (Wilstaett, Germany) ferritin, sTfR, RBP, CRP and AGP were analysed using the sandwich ELISA method as described by Erhardt *et al*. [18].

### Statistical analysis

Two staff members double entered the data into a database (Access 2003, Microsoft) using EpiInfo version 3.5.4 (CDC, Atlanta, USA). The database crosscheck was performed using the Data Compare tool of EpiInfo. Any discrepancies between both entries were removed by referring to the original data sheets. The descriptive distribution of micronutrients involving the mean, median, SD, lower quartile, upper quartile of micronutrient serum values was calculated, as well as the prevalence of micronutrient deficiencies.

Anthropometric z-scores were calculated using a readily available macro-statistical package developed by a technical group of WHO [19] that incorporated the WHO child growth standards for children aged below five and children aged 5–19 years [20]. Children were classified as underweight, stunted and wasted if their z-scores were less than or equal to -2 standard deviation (SD) below the WHO median of standards for age and sex. Children were classified as overweight and obese using z-score cut-off points of +2SD and +3SD, respectively. Since weight-for-age reference data is not available for older children (above 10 years of age), due to its inability to distinguish relative height and body mass during pubertal growth spurt [20], we categorized children’s age into two groups during the analysis: below (hereinafter referred to as “younger children”) and above 10 years of age (hereinafter referred to as “older children”). For BMI, four categories were created: underweight, (BMI<18.50 kg/m^2^), normal (BMI = 18.50–24.90 kg/m^2^), overweight (BMI = 25.00–29.90 kg/m^2^) and obese (BMI≥30.00 kg/m^2^). Both z-scores and BMI were analysed as continuous and categorical variables.

Severity of anemia was determined according to the WHO age-specific recommended cut-off values [8]. Anemia was categorized into four groups: no anemia (≥115 g/l for 5–11 years and ≥120 g/l for 12–14 years), mild anemia (110–114 g/l for 5–11 years and 110–119 g/l for 12–14 years), moderate anemia (80–109 g/l for 5–11 years and 80–109 g/l for 12–14 years) and severe anemia (<80 g/l for both 5–11 and 12–14 years). Using a published algorithm from Weiss *et al*. [21], anemia was further classified into three groups: iron-deficiency anemia (IDA), anemia of chronic disease (ACD) and IDA+ACD. Briefly, anemic participants were classified as having IDA when they had a ferritin concentration below 30 ng/ml. Anemic participants were classified as having ACD when their ferritin concentration was between 30 and 100 ng/ml and their ratio of sTfR to the log of serum ferritin was below 1 or when their ferritin concentration was above 100 ng/ml. And finally, anemic participants were classified as having ACD with true IDA when they had a ferritin concentration between 30 and 100 ng/ml and their ratio of sTfR to the log of serum ferritin was above 2.

All statistical analyses were done using R version 3.6.1 (R Core Team; R Foundation for Statistical Computing; Vienna, Austria). We applied a bivariate logistic regression to ascertain the association between known risk factors and the outcome. The outcomes of the regression analyses were frequency and distribution of nutritional indicators, anemia and micronutrient deficiency. The risk factors included in the analysis were age, sex, socio-economic status, household condition (type of floor, wall and roof), presence and location (inside or outside the house) of a toilet facility, source of drinking water, shoe wearing habit and animal ownership. In addition, we included the following parasitic infections: *P*. *falciparum*, *S*. *haematobium*, *S*. *mansoni*, *Entamoeba* *histolytica*/*Entamoeba* *dispar*/*Entamoeba* *moshkovskii* (molecular characterization was not carried out, therefore, we could not distinguish between these three species), *G*. *lamblia* and filarial infections.

The mixed-effect logistic regression models for nutritional indicators, micronutrient deficiencies, serum biomarkers and anemia were developed, using an automated stepwise backward elimination procedure. We adjusted for age, sex, socio-economic status, CRP, AGP and school clustering effect. Crude odds ratios (cORs), adjusted odds ratios (aORs) and their corresponding 95.00% CIs were calculated. To compare proportions of schoolchildren with malnutrition by sex and school location, we used χ2 statistics. All results were considered significant at the level of *p*<0.05.

## Results

### Anthropometric and nutritional indicators

Table 1 presents the demographic and macro-nutritional characteristics of participants. The study population was homogeneous in terms of participants’ sex (48.70% girls and 51.30% boys) and age (mean 9.67 ± 1.81 years for girls and 9.89 ± 1.59 for boys). Overall, the population distribution of weight-for-age, height-for-age and BMI-for-age z-scores was the same between boys and girls (*p*>0.05). Nearly half of all the schoolchildren were malnourished (n = 220, 46.61%) and only one was overweight. Stunting (23.88%) was the most prevalent type of malnutrition followed by underweight (18.94%) and wasting (16.32%). Most malnourished children (67.20%) were not diagnosed with any type of parasitic infection (S1 Table). Also, there was no significant association between nutritional status (nourished vs malnourished) and parasitic infections (infected vs non-infected) among schoolchildren (*p*>0.05) (S1 Table).

**Table 1:** Frequency of nutritional indicators and anemia among schoolchildren in Kikwawila and Kiberege wards, Tanzania, stratified by sex and location of the school.

Category	Girls	Boys	*p*-value	Least rural schools	Most rural schools	*p*-value
Age (years)						
Mean ± SD	9.67±1.81	9.74±1.79		9.58±1.90	9.89±1.59	
Median (range)	10 (6–12)	10 (6–12)		10 (6–12)	10 (9–11)	
Height (cm)						
Mean ± SD	130.20±11.83	128.37±9.27		129.80±11.06	128.2±9.79	
Median (range)	131 (99–166)	128 (106–153)		130 (121–137)	129 (122–135)	
Weight (kg)						
Mean ± SD	26.54±6.25	25.28±4.93		25.78±6.03	25.91±4.98	
Median (range)	26 (15.50–47.30)	25 (17.5–51.50)		24.5 (20.50–29.50)	25.05 (22.30–28.62)	
Haemoglobin			0.02*			0.05*
Mean ± SD	134.9±37.06	128.3±16.06		133.40±34.85	128.60±13.35	
Anemia			0.31			0.67
^+^Mild (%)	17 (8.33)	23 (10.55)		21 (8.24)	19 (11.38)	
^+^Moderate (%)	7 (3.43)	10 (4.50)		12 (4.71)	5 (3)	
^+^Normal	180 (88.24)	185 (84.86)		222 (87.06)	143(85.63)	
HAZ			0.11			0.16
Mean ± SD	-1.08±1.38	-1.25±1.20		-0.98±1.17	-1.46±1.42	
Stunted (%)	50 (22.94)	54 (24.66)		48 (18.39)	56 (33.53)	
WAZ			0.15			0.9
Mean ± SD	-1.01±1.17	-1.16±0.92		-0.98±1.17	-1.14±0.98	
Underweight (%)	27 (13.04)	25 (12.20)		32 (19.16)	20 (19.8)	
BAZ			0.135			0.6
Mean ± SD	-0.79±1.25	-0.96±1.17		-1.06±1.09	-0.72±1.25	
Wasted (%)	32 (15.31)	37 (16.89)		43 (16.86)	25 (14.97)	
Overweight (%)	0	1		1	0	

**Note:** BAZ, BMI-for-age Z-scores; HAZ, Height-for-age Z-score; WAZ, Weight-for-age Z-scores; SD, Standard deviation.

*Two-sample t-test.

^+^Haemoglobin deficiency categorized by WHO.

### Prevalence of anemia

The overall mean and median hemoglobin concentration among the 427 participants (90.10% of all participants) who provided a venous blood sample were 133.6±51.10 g/l and 128 g/l, respectively. The mean distribution of hemoglobin concentration significantly differed between boys and girls (*p* = 0.02); girls had a higher hemoglobin concentration (134.9±37.06 g/l) than boys (128.3±16.06 g/l). Most schoolchildren (86.36%) had a normal hemoglobin concentration during diagnosis. Only 13.51% of schoolchildren were diagnosed with hemoglobin deficiency (anemia), with no significant difference between sexes (43 boys and 24 girls, *p* = 0.06). Of these, 9.62% and 4.08% of schoolchildren had mild and moderate anemia, respectively. None of the schoolchildren was diagnosed with severe anemia. Of the 67 anemic schoolchildren, a higher proportion were IDA (n = 26; 48.15%), followed by IDA+ACD (n = 11; 20.37%) and ACD (n = 4; 7.40%). About 13% and 19% of schoolchildren had a decreased size (MCV) and weight (MCHC) of haemoglobin, respectively, which indicates microcytic anemia.

We used ferritin, transferrin and sTfR proteins as biomarkers for diagnosis of iron-deficiency anemia in schoolchildren. About 35% and 12% schoolchildren had above-normal levels of sTfR and low levels of ferritin, respectively. Schoolchildren with elevated levels of sTfR were approximately three times (aOR: 2.51, 95% CI: 1.15–5.63) more likely to be diagnosed with anemia (Table 3). Male students and those infected with *P*. *falciparum* were more likely to be diagnosed with elevated levels of sTfR. Owning a chicken or a dog was associated with lower likelihood of being diagnosed with lower levels of ferritin (aOR: 0.40, 95% CI: 0.17–0.96) and transferrin (aOR: 0.25, 95% CI: 0.06–0.85). As markers of inflammation, CRP and AGP levels were elevated above normal cut-off points in 13.61% and 11.07% of schoolchildren, respectively.

**Table 2:** Levels of hemoglobin, complete blood count, serum micronutrients, anemia and inflammatory markers among schoolchildren in Kikwawila and Kiberege wards, Tanzania.

Category	Unit	Cut-off values	Group	References	N	Mean±SD	Median (IQR)	Deficiency	
								N	%
**Anemia**									
Hemoglobin	g/l	<115	5 to 11 years	[8,62]	422	131.49±28.43	128 (120, 135)	57	13.51
		<120	12 to 14 years						
MCV	fl	61.20–85.10	6–10 years	[63,64]	446	78.20±9.10	79.70 (76.00, 82.60)	53	12.68
		70.09–95.34	>10 years						
MCH	pg/cell	20.30–29.20	6–10 years	[63,64]	423	27.08±2.80	27.30 (25.50, 28.70)	67	18.82
		22.85–32.78	>10 years						
MCHC	g/dl	31.30–36.80	6–10 years	[63,64]	423	36.15±1.63	33.90 (33.1, 34.70)	20	4.72
		30.67–36.33	>10 years						
**Anemia and inflammatory markers**									
sTfR	mg/l	>8.30	< 6 years	[65]	418	8.03±2.77	7.28 (6.36, 8.85)	145	34.69
Ferritin	μg/l	<15	Non-malaria	[66]	406	50.19±37.97	42.10 (24.80, 62.35)	50	12.32
		<30	Malaria						
Transferrin	g/l	>3.60	< 6 years	[65]	435	2.94±0.54	2.90 (2.60, 3.20)	43	9.89
AGP	g/l	1	NS	[67,68]	372	0.68±0.32	0.61 (0.46, 0.81)	57*	13.57^+^
CRP	mg/l	>5	NS	[67,68]	311	2.69±7.20	0.47 (0.21, 1.38)	33*	11.07^+^
**Micronutrient deficiency**									
Vitamin A	μM/l	0.70–1.05	Marginal	[10]	435	1.32±0.57	1.19 (0.96, 1.57)	151	34.71
		<0.70	Severe						
RBP	μmol/l	<0.70	All years	[69]	420	0.99±0.31	0.96 (0.81, 1.13)	41	10.47
Vitamin B12	pmol/l	<150	All years	[70]	432	322.90±142.4	297 (227.5, 402)	38	8.79
Zinc	μg/dl	65	< 10 years	[71]	433	106±3.81	100.74 (91.14, 114.45)	8	1.85
		66 (female)	≥ 10 years						
		70 (male)							
Folate	nmol/l	<10	All years	[70]	408	29.40±8.92	28.70 (22.4, 36.15)	2	0.5

**Note:** AGP, alpha-1- acid glycoprotein; CRP, C-reactive protein; IQR, Interquartile range; MCH, mean corpuscular hemoglobin, MCHC, mean corpuscular hemoglobin concentration, MCV, mean corpuscular volume; N, Number of observations; NS, Not specified; RBP, retinol-binding protein; SD, Standard deviation; sTfR, soluble transferrin receptor.

*Number of schoolchildren found with inflammation.

^+^Percentage of schoolchildren found with inflammation.

### Micronutrient deficiencies

Nearly half of the participants (n = 184, 45.60%) were diagnosed with at least one micronutrient deficiency. The most prevalent deficiency in schoolchildren was vitamin A (34.71%); 27.35% and 7.36% of children had marginal and severe vitamin A deficiency, respectively. We also found that 10.50% of our participants had a low concentration of RBP, and that the concentration of RBP was positively correlated to that of vitamin A (p<0.001) (S1 Fig). Additionally, 8.79% of schoolchildren had a vitamin B12 deficiency with a high variability in the mean concentration (322.90±142.40 pmol/l) among screened children.

Schoolchildren attending the most rural schools were five times more likely to be diagnosed with at least one micronutrient deficiency (aOR: 5.04, 95% CI: 2.38–11.44). We also found that older children were less likely to be diagnosed with a micronutrient deficiency (aOR: 0.48, 95% CI: 0.25–0.90). (Table 3). Children with elevated levels of CRP were approximately four times more likely to be diagnosed with low levels of RBP and vitamin A deficiency. Only 1.87% and 0.50% of schoolchildren were found deficient of zinc and folate, respectively.

**Table 3:** Risk factors for nutritional status, anemia and selected micronutrient deficiency in schoolchildren in the Kikwawila and Kiberege wards, Tanzania.

Morbidity indicator	Significant association	cOR (95% CI)	aOR (95% CI)
**Nutritional status**			
Stunting	Micronutrient levels		
	Vitamin B12 deficiency	3.11 (1.30–7.36)	3.33 (1.26–8.86)
	Demographic variables		
	Most rural schools	2.43 (1.38–4.33)	3.26 (1.69–6.44)
	10–12 years of age	3.08 (1.74–5.54)	3.46 (1.87–6.55)
Underweight	Elevated sTfR conc	2.14 (0.94–4.93)	3.90 (1.35–12.36)
Wasting	Low ferritin conc	0.15 (0.01–0.72)	0.07 (0.004–0.46)
	Zinc deficiency	2.58 (0.35–13.66)	14.10 (1.22–161.24)
	Anemia	1.53 (0.61–3.49)	3.16 (1.06–9.25)
	10–12 years of age	2.37 (1.25–4.60)	4.98 (2.20–12.04)
**Anemia**			
Low hemoglobin conc	Anemia markers		
	Elevated sTfR conc	3.18 (1.56–6.69)	2.51 (1.15–5.63)
	Low MCV conc	4.47 (1.43–13.05)	4.30 (1.23–14.36)
**Anemia markers**			
Low ferritin conc	*P*. *falciparum*	5.78 (1.99–15.85)	10.40 (2.88–40.53)
	Owning chicken	0.54 (0.25–1.21)	0.40 (0.17–0.96)
Elevated sTfR conc	*P*. *falciparum*	4.19 (1.60–12.26)	3.59 (1.27–11.23)
	Male students	2.32 (1.42–3.85)	2.09 (1.25–3.55)
Low transferrin conc	Most rural schools	5.15 (2.17–13.62)	6.47 (2.52–18.29)
**Micronutrient deficiency**			
Vitamin A deficiency	Elevated CRP conc	3.66 (1.70–8.25)	3.60 (1.53–8.94)
	10–12 years of age	0.53 (0.31–0.88)	0.46 (0.26–0.79)
	Male students	1.62 (0.99–2.64)	1.69 (1.02–2.84)
Low RBP conc	Elevated CRP conc	4.04 (1.61–9.62)	3.80 (1.34–10.47)
Any micronutrient deficiency	Most rural schools	3.02 (1.62–5.95)	5.04 (2.38–11.44)
	10–12 years of age	0.56 (0.33–0.98)	0.48 (0.25–0.90)

**Note:** aOR, adjusted odds ratio; CI, confidence interval; cOR, crude odds ratio; conc, concentration; CRP, C-reactive protein; *E*. *histolytica*, *Entamoeba histolytica; E*. *dispar*, *Entamoeba dispar*; *E*. *moshkovskii*, *Entamoeba moshkovskii*; MCV, mean corpuscular volume; *P*. *falciparum*, *Plasmodium falciparum*; RBP, Retinol binding protein; sTfR, soluble transferrin receptor.

### Micronutrient deficiency, nutritional status and underlying risk factors

Stunting among schoolchildren was associated with attending one of the most rural schools (aOR: 3.26, 95% CI: 1.69–6.44), older children (aOR: 3.46, 95% CI: 1.87–6.55) and having vitamin B12 deficiency (aOR: 3.33, 95% CI: 1.26–8.86) (Table 3). Wasting was more common among schoolchildren diagnosed with anemia (aOR: 3.16, 95% CI: 1.06–9.25) and older children (aOR: 4.98, 95% CI: 2.20–12.04). Children with vitamin A deficiency had a higher odds of wasting (aOR: 2.31, 95% CI: 1.16–4.6). Schoolchildren who had elevated levels of sTfR were more likely to be underweight (aOR: 3.90, 95% CI: 1.35–12.36) compared to those with low sTfR concentration.

## Discussion

The current knowledge on the prevalence of malnutrition, anemia, micronutrient deficiency and their potential association to parasitic infections is scarce in Sub-Saharan Africa, and especially lacking in Tanzania. Our study is the first, in the last three decades, to investigate their distribution and underlying risk factors among schoolchildren in Tanzania. We found that, in this research setting, a high percentage of children were stunted, underweight, wasted, anemic and with micronutrient deficiencies.

### Anthropometric measurements and nutritional status

Anthropometry is a commonly used quantitative measurement to determine the growth trend of children and population’s undernutrition in the form of stunting, wasting and underweight [22]. Stunting is a result of poor development and growth during childhood, and it is often caused by poor nutrition and repeated parasitic infections [23]. Despite a 30% decrease in stunting over the last 25 years nationwide [24], stunting still remains a significant health problem in Tanzania, which is among the 14 nations with the highest global burden of stunting [25]. The prevalence of stunting in our participants (23.90%) was not as high as the reported national prevalence (34.63%) [24]. This difference between our study and the national prevalence might be explained by clustered regional variations; it is known that the south-western regions have higher prevalence of stunting than the north-eastern regions of Tanzania [26]. Our finding of higher prevalence of stunting among schoolchildren attending the most rural schools is in line with a study in Ghana [27]. Similarly, in our previous publication based on the data collected during the current cross-sectional survey, we found that the most rural schools had the highest prevalence of parasitic infections [16]. Environmental factors and low socio-economic status are among the driving factors for parasitic infections, which may have consequently led to an increase of stunting in the most rural schools. Despite not finding a significant association between parasitic infections and nutritional indicators in this study (S1 Table), the impact of parasitic infections on stunting [28], wasting [29] and underweight [30] cannot be understated. Parasitic infections have been reported to play an important role in malnutrition by causing protein-energy malnutrition, anemia and physical complications as a result of increased nutrient wastage, excessive blood loss, intestinal obstruction and rectal prolapse [30,31]. Additionally, as noted by UNICEF [32], child malnutrition is a complex, multisectoral problem resulting from a combination of inadequate dietary intake (protein energy and micronutrients) and infection. The underlying causes of malnutrition can be clustered into three groups: such as insufficient access to adequate and quality food, poor maternal and child care practice, and inadequate prevention and control of diseases.

Often malnutrition is closely associated with anemia owing to similar causal factors such as poor maternal nutrition, infectious diseases and inadequate complimentary feeding at an early age [8,33]. In our study, schoolchildren diagnosed with anemia were three times more likely to be diagnosed with wasting too. Our results are in line with several studies done in Kenya [34] and West Africa [35], which pinpoint the underlying need to incorporate a nutritional improvement package in health promotion programmes.

### Anemia

Anemia continues to be the major public health problem worldwide [36,37] that can cause a reduction in growth height, school attendance and loss of earning [38]. We observed a relatively low prevalence of anemia, which can be attributed to the high mean concentration of hemoglobin and, perhaps, even the low prevalence of parasites among participants. Mean and median hemoglobin concentrations were higher in our study, compared to two other studies conducted in the Tanga region, Tanzania [39,40], but consistent with two studies conducted in Ethiopia [41,42]. The higher concentration of hemoglobin in girls compared to boys found in our study was in line with a study done in West Africa [43] and might be partly explained by the mean and median age of girls recruited being below 11 years old, an age where most Tanzanian girls have not yet reached their menarche [44,45].

Apart from hemoglobin concentration measurements, several biological markers such as ferritin, transferrin and sTfR can be useful proxies for assessing changes in body iron status and anemia [8]. Normally, the levels of sTfR rise in response to the decrease of iron concentration in the blood, indicating anemia [46]. The higher likelihood of schoolchildren with elevated levels of sTfR to be found anemic in our study (Table 3), provides additional evidence that sTfR might be a good biomarker for anemia diagnosis. Ferritin, on the other hand, was not found to be a good biomarker for diagnosis of anemia in our study, since we did not observe the expected low ferritin levels among anemic children [47]. The higher ferritin concentration levels in our study population might be due to inflammation and/or acute infection that we did not diagnose [8]. Parasitic infections have also been known to negatively affect the levels of serum iron and other micronutrients through a subtle worsening of digestion and absorption, chronic inflammation of the gut and loss of nutrients. In Tanzania, a high prevalence of anemia in children (59.00%) and women of reproductive age (41.00%) [48] has been found to be highly associated with malaria and STH infections [49]. The ability of *P*. *falciparum* to cleave and endocytose ferritin, might explain the low levels of ferritin observed in our study [50,51].

### Association between micronutrient deficiency and nutritional status

Micronutrient deficiency plays a crucial role in human malnutrition, which further exacerbates a vicious cycle of physical, cognitive and socio-economic underdevelopment of already under-privileged communities. In this study, we observed a large variability in micronutrient deficiencies ranging from a prevalence of only 0.50% in the case of folate deficiency up to a prevalence of 34.71% in the case of vitamin A deficiency. In Tanzania, vitamin A deficiency is a severe public health problem [10] with more than 20.00% of the children under five years of age being diagnosed with RBP concentrations below 0.70 μmol/L [48]. The prevalence of vitamin A deficiency varies widely in-country, ranging from 26.60% in the Singida region to 57.30% in North Pemba.

In addition, we observed a high variability of vitamin B12 concentration (Table 2), which has been previously reported [52], as well as the negative effect that vitamin B12 deficiency has on nutritional status expressed as stunting (Table 3) [53]. These findings are in line with studies done in Colombia and Nepal, where vitamin B12 deficiency was strongly associated with stunting [54] and low socio-economic status [53], respectively.

The prevalence of zinc deficiency in our study (1.85%) was surprisingly low compared to the national prevalence, where zinc deficiency is currently known to affect up to 70% of children under five years of age [55]. One possible explanation might be related to possible hemolysis during sample collection and/or processing. Since red blood cells contain large amounts of zinc, their hemolysis can lead to a release of zinc into the serum, causing an increase of the serum zinc concentration, which interferes with an accurate measurement [56]. Zinc plays a vital role in protein synthesis and cellular growth during childhood. Zinc deficiency can ultimately lead to poor weight and height gain, which affects the overall growth trend [57]. The association between zinc deficiency and wasting found in our study was in line with several other studies, including a meta-analysis [58,59].

### Limitations

Our study has a few limitations. First, this cross-sectional study was conducted in nine primary schools located in only two wards in south-eastern Tanzania. Therefore, results from this study may not be generalizable to a larger population. Second, because this survey was a one-time cross-sectional survey we cannot infer the causality between malnutrition, anemia, micronutrient deficiency, and parasitic infections. Third, we did not collect data pertaining to the participant’s dietary habits and the diversity of their diets. This information may have helped us understand individual and household macro- and micronutrient intake adequacy, which might have, in turn, helped explain the nutritional status of our participants. Fourth, since this is a school-based study and participants were selected in schools, there may have been some selection bias that is important to highlight; it could be that girls attending school are generally healthier and wealthier than boys attending school, which could have some influence on our results. Finally, it should be noted that, given the lack of universal cut-off values, our selection of cut-off values for some micronutrients could influence our results.

## Conclusions

Our study revealed that malnutrition, anemia, micronutrient deficiency and parasitic infections are still highly prevalent among schoolchildren living in the rural areas of south-eastern Tanzania. Parasitic infections and micronutrient deficiencies are significantly associated with anemia and nutritional indicators. To effectively control and, consequently, eliminate malnutrition, anemia and micronutrient deficiencies in these settings, there is a dire need of nutritional, disease control, water, sanitation and hygiene (WaSH) programs that focus on improving dietary quality, increasing production of nutritious food and consumption of animal source food, education on optimal dietary practices, early treatment, immunization and improving WaSH facilities and practices [32,60,61]. Dietary improvement may be an ideal and sustainable approach to increase multiple micronutrient intake, target a large group population (including non-targeted, poor and undernourished groups) and to preserve sensory qualities of food [60]. In addition, unique tailoring of these programs to suit local needs, such as increasing availability and consumption food and/or supplements containing iron, vitamin A and B12, which have positive impact in growth and development cannot be understated.

As described in our previous publication [16], more than a quarter of the participating schoolchildren were infected with at least one parasite. These parasitic infections were significantly associated with the use of unimproved sanitation facilities. We believe adequate control measures, such as improved access to WaSH, health education and continuation of use of insecticide-treated net and periodic deworming could eliminate parasitic infections in this region.

## Supporting information

S1 Fig Correlation between vitamin A concentration and RBP concentration. Common regression lines show r = 0.58, *p*<2.2e-16. Note: RBP, Retinol binding protein.
(TIF)

S1 Table Presence of parasitic infections *versus* nutritional indicators in schoolchildren in Kikwawila and Kiberege wards, Tanzania. Note: *zero outcome; E. histolytica, Entamoeba histolytica; E. dispar, Entamoeba dispar; E. moshkovskii, Entamoeba moshkovskii; P. falciparum, Plasmodium falciparum; S. haematobium, Schistosoma haematobium; S. mansoni, Schistosoma mansoni.
(DOCX)

S1 Text Standard operating procedure assessing anthropometric parameters in schoolchildren of Kikwawila and Kiberege wards, Tanzania. (DOC)
