# Supplementary figures and images for: Malnutrition, anemia, micronutrient deficiency and parasitic infections among schoolchildren in rural Tanzania
Source: PLoS Negl Trop Dis. 2022 Mar 4;16(3):e0010261. doi: 10.1371/journal.pntd.0010261 (PMC8926280; doi:10.1371/journal.pntd.0010261)

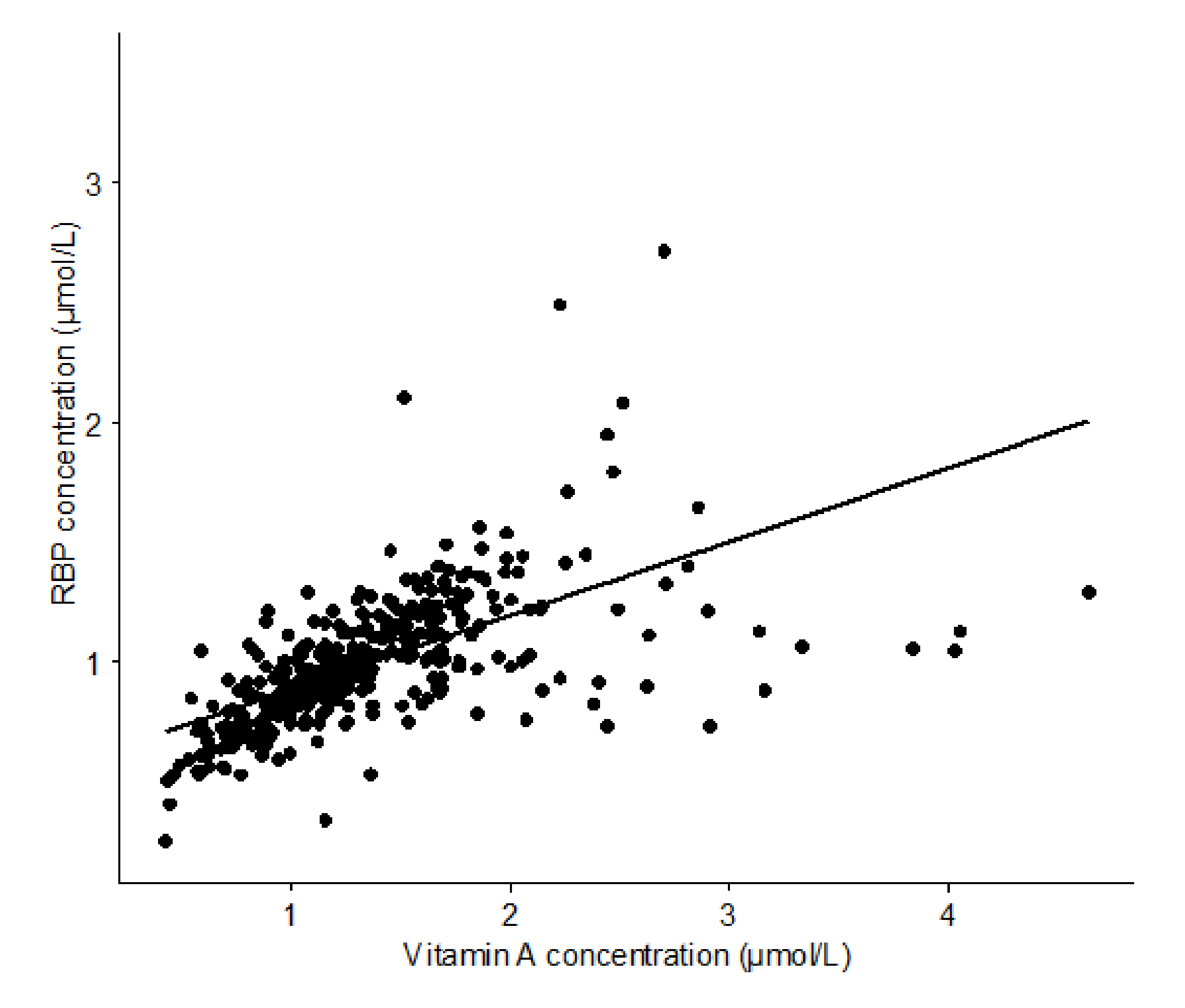

Supplement: S1 Fig — Common regression lines show r = 0.58, p<2.2e-16. Note: RBP, Retinol binding protein. (TIF) [file pntd.0010261.s001.tif]
