# Supplementary material for: Malnutrition, anemia, micronutrient deficiency and parasitic infections among schoolchildren in rural Tanzania
Source: PLoS Negl Trop Dis. 2022 Mar 4;16(3):e0010261. doi: 10.1371/journal.pntd.0010261 (PMC8926280; doi:10.1371/journal.pntd.0010261)
Supplement: S1 Table — Note: *zero outcome; E. histolytica, Entamoeba histolytica; E. dispar, Entamoeba dispar; E. moshkovskii, Entamoeba moshkovskii; P. falciparum, Plasmodium falciparum; S. haematobium, Schistosoma haematobium; S. mansoni, Schistosoma mansoni. (DOCX) [file pntd.0010261.s002.docx]

S1 Table: Presence of parasitic infections *versus* nutritional indicators in schoolchildren in Kikwawila and Kiberege wards, Tanzania.

|  | **Nutritional indicators** | | | | | | | | | |
| --- | --- | --- | --- | --- | --- | --- | --- | --- | --- | --- |
|  | **Wasting** | | ***p*-value** | **Stunting** | | ***p*-value** | **Underweight?** | | ***p*-value** |  |
|  | **Yes** | **No** |  | **Yes** | **No** |  | **Yes** | **No** |  |  |
| *E. histolytica/ E. dispar/*  *E. moshkovskii* |  |  | 0.79 |  |  | 0.28 |  |  | 0.31 |  |
| Infected | 10 | 56 |  | 20 | 46 |  | 6 | 38 |  |  |
| Not infected | 59 | 299 |  | 83 | 275 |  | 45 | 177 |  |  |
| *P. falciparum* |  |  | 0.86 |  |  | 0.34 |  |  | 0.29 |  |
| Infected | 4 | 19 |  | 8 | 15 |  | 4 | 7 |  |  |
| Not infected | 65 | 340 |  | 96 | 309 |  | 48 | 209 |  |  |
| *S. haematobium* |  |  | * |  |  | 1.00 |  |  | * |  |
| Infected | 0 | 3 |  | 1 | 2 |  | 0 | 2 |  |  |
| Not infected | 69 | 356 |  | 103 | 322 |  | 52 | 214 |  |  |
| *S. mansoni* |  |  | 0.12 |  |  | 0.41 |  |  | 0.83 |  |
| Infected | 4 | 48 |  | 15 | 37 |  | 6 | 30 |  |  |
| Not infected | 65 | 311 |  | 89 | 287 |  | 46 | 186 |  |  |

Note: *zero outcome; *E. histolytica, Entamoeba histolytica*; *E. dispar*, *Entamoeba dispar*; *E. moshkovskii*, *Entamoeba moshkovskii*; *P. falciparum, Plasmodium falciparum*; *S. haematobium, Schistosoma haematobium*; *S. mansoni, Schistosoma mansoni*
