# Supplementary material for: Malnutrition, anemia, micronutrient deficiency and parasitic infections among schoolchildren in rural Tanzania
Source: PLoS Negl Trop Dis. 2022 Mar 4;16(3):e0010261. doi: 10.1371/journal.pntd.0010261 (PMC8926280; doi:10.1371/journal.pntd.0010261)
Supplement: S1 Text — (DOC) [file pntd.0010261.s003.doc]

**Standard operating procedure (SOP) for schoolchildren anthropometric measures**

**Purpose**:

This SOP describes how to measure the two anthropometric measurements: height and weight

**Procedure for measuring height**:

For measuring the child’s height, the following equipment is required:

- **Wall mounted stadiometer roll-ruler**

1. Place the child on his/her back against the wall.
2. Have the child stand with feet slightly apart and back as straight as possible. The heels, buttocks and shoulder blades should touch the wall.
3. Have the child look straight ahead with head erect.
4. Lower the base of the stadiometer to the head until it firmly touches the crown of the child’s head.
5. Read the measurement at eye level where they appear in the reading window.
6. Read measurement on the under edge to the nearest of 1 cm.
7. Record the measurement on the provided form.

**Procedure for measuring weight:**

For measuring the child’s weight, a **scale** is required:

1. Check that the child removes shoes, backpack and heavy/bulky outer clothing such as coat, jacket, or bulky sweatshirt.
2. Have the child step onto the center of the scale platform with feet slightly apart for better balance.
3. Read the weight when the number is stable to the nearest 0.1 kg.
